# Anticancer Therapy and Mortality of Adult Patients with Hematologic Malignancy and COVID-19: A Systematic Review and Meta-Analysis

**DOI:** 10.3390/life13020381

**Published:** 2023-01-30

**Authors:** Wen-Li Lin, Thi-Hoang-Yen Nguyen, Li-Min Wu, Wen-Tsung Huang, Shih-Bin Su

**Affiliations:** 1 Center for Quality Management, Chi Mei Medical Center, Liouying, Tainan 71004, Taiwan; 2 School of Nursing, Kaohsiung Medical University, Kaohsiung 80708, Taiwan; 3 Department of Environmental and Occupational Health, National Cheng Kung University, Tainan 704302, Taiwan; 4 Department of Medical Research, Kaohsiung Medical University Hospital, Kaohsiung 80756, Taiwan; 5 Division of Hematology and Oncology, Department of Internal Medicine, Chi Mei Medical Center, Liouying, Tainan 71004, Taiwan; 6 Department of Occupational Medicine, Chi Mei Medical Center, Tainan 71004, Taiwan

**Keywords:** anticancer therapy, mortality, hematologic malignancy, COVID-19, systematic review, meta-analysis

## Abstract

Coronavirus disease 2019 (COVID-19) might affect cancer treatment outcomes. This systematic review and meta-analysis identified the prognostic predictors of adult patients with hematologic malignancies and COVID-19, and evaluated the effect of anticancer therapy on mortality. We performed a literature search of electronic databases and identified additional studies from the bibliographies of the articles that were retrieved. Two investigators independently extracted data according to the Preferred Reporting Items for Systematic Reviews and Meta-analyses (PRISMA) reporting guidelines. We evaluated study quality using the Newcastle–Ottawa Scale and performed a meta-analyses in order to evaluate the effect of anticancer therapy on mortality among adult patients with hematologic malignancies and COVID-19. Heterogeneity was assessed with the *I*^2^ statistic. The meta-analysis included 12 studies. The overall mortality rate was 36.3%. The pooled risk difference (RD) in mortality between patients receiving and not receiving anticancer therapy was 0.14 (95% confidence interval [CI]: 0.02–0.26; *I*^2^ = 76%). The pooled RD in mortality associated with chemotherapy was 0.22 (95% CI: 0.05–0.39; *I*^2^ = 48%), and with immunosuppression was 0.20 (95% CI: 0.05–0.34; *I*^2^ = 67%). In the subgroup analyses, anticancer-therapy-associated mortality was higher in females (RD = 0.57; 95% CI: 0.29–0.85; *I*^2^ = 0%) than in males (RD = 0.28; 95% CI: 0.04–0.52; *I*^2^ = 0%). Among patients with hematologic malignancies and COVID-19, those receiving anticancer therapy had a higher mortality risk, regardless of sex. The mortality risk was higher in females than in males. These results indicate that caution should be exercised when administering anticancer therapy to patients with hematologic malignancies and COVID-19.

## 1. Introduction

Caused by severe acute respiratory syndrome coronavirus 2 (SARS-CoV-2), the coronavirus disease 2019 (COVID-19) pandemic is still ongoing in many countries worldwide. The World Health Organization’s most recent estimate shows that, as of September 2022, there have been >600 million COVID-19 cases worldwide, with >6 million deaths [1]. COVID-19 symptoms range from being undetectable to causing death, but most commonly include fever, dry cough, and fatigue. Severe illness is more common in patients with cancer or certain other underlying medical conditions [2].

Patients with cancer are a vulnerable population in the COVID-19 pandemic. Because of their underlying illness, poor nutrition, and treatment-related side effects, patients with cancer had a higher risk of contracting COVID-19 than those without cancer [3,4]. In addition, once infected with SARS-CoV-2, patients with cancer had poorer outcomes. A high neutrophil count has frequently been seen in patients with cancer. Influenza was associated with a higher mortality rate in patients with hematologic and solid cancers [5]. Comparisons between patients with and without hematological malignancy confirmed the vulnerability of those with hematological malignancy in the current COVID-19 pandemic. Furthermore, rhinovirus is associated with a significant increase in mortality when present before hematopoietic cell transplant [5]. With the onset of the COVID-19 pandemic, there has been a significant global effort to understand the effects of SARS-CoV-2 infection on cancer patients [6]. Studies have shown that infections are more common in patients with cancer, particularly hematologic malignancies, than in patients without cancer [7,8]. Moreover, some studies have reported that hematologic malignancies are associated with severe COVID-19 morbidity [9,10,11].

The care of cancer patients infected with SARS-CoV-2 has been the subject of several guidelines and review studies [12,13,14]. Two large US studies reported an association between increased mortality and cytotoxic chemotherapy in the previous month or three months [15,16]. Evidence suggests that immune checkpoint inhibitors might even help to treat viral infections by preventing or reducing T-cell exhaustion. In this context, the correct timing of treatment might be essential. Nevertheless, some cancer patients who are treated with immune checkpoint inhibitors experience autoimmune-related side effects that require immunosuppressive therapies, which may promote a severe course of SARS-CoV-2 infection [17].

Data on COVID-19-associated morbidity and mortality in cancer patients receiving anticancer therapy are less clear and often conflicting [18]. It is critical to aggregate data in order to obtain more accurate estimates of the COVID-19-associated risks [19]. In particular, some recent studies reported findings that differ from the previous meta-analysis [20]., and there have been no further meta-analyses specific to patients with hematologic malignancies up to 2022. This study aimed to perform a systematic literature review and meta-analysis in order to quantify the impact of anticancer therapy on patients with hematologic malignancies and COVID-19.

## 2. Materials and Methods

### 2.1. Literature Search

**Figure 1:**
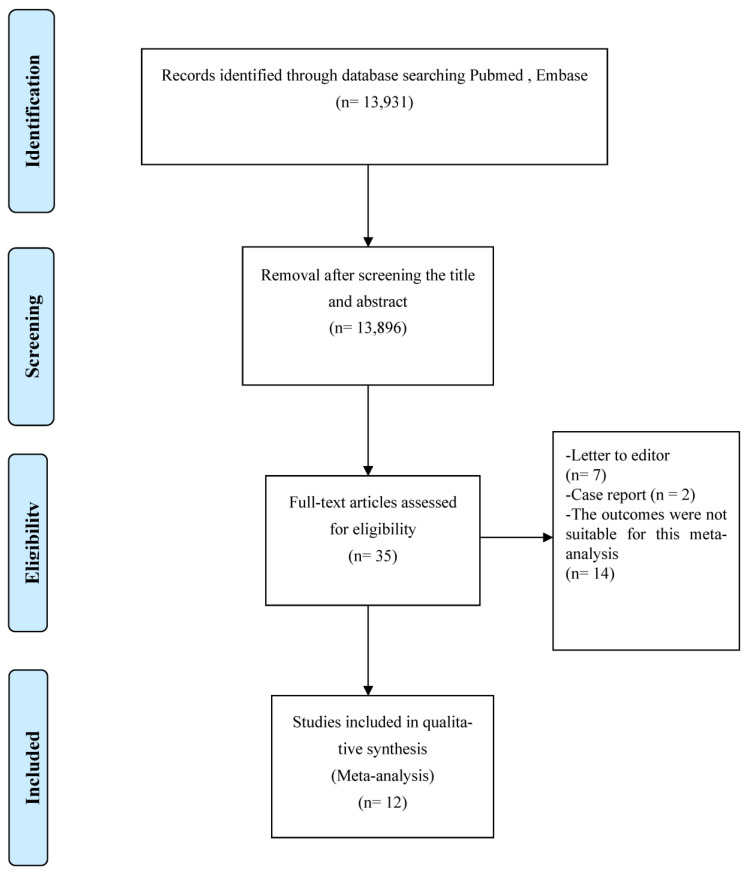
Flow diagram of the literature search.

This systematic review and meta-analysis were performed according to the Preferred Reporting Items for Systematic Reviews and Meta-analyses (PRISMA) guidelines (Supplementary Materials). Two authors independently searched the PubMed, Embase, and Web of Science databases for articles that were published from inception of the database up to September 2022. They used the keywords “coronavirus”, “COVID-19”, “SARS-CoV-2”, and “2019-nCoV”, with “hematologic malignancy”, “leukemia”, and “lymphoma” in order to identify articles on patients with hematologic malignancies and COVID-19. In order to identify articles regarding the relevant outcome, they also included the keywords “mortality”, “survival”, and “death”. Figure 1 presents a flowchart detailing the literature search performed to identify the articles used in the systematic review and meta-analysis.

### 2.2. Inclusion and Exclusion Criteria

All the case–control and cohort studies reporting data on adults (aged > 18) with a SARS-CoV-2 infection and hematologic malignancies were included. All the review articles, case reports, letters to the editor, and other literature whose outcomes were unsuitable for this meta-analysis were excluded.

### 2.3. Data Extraction

The following information was recorded: publication authors, study region, publication year, primary diagnoses, patient numbers, patient age, sex distribution, anticancer therapy type (including bone marrow transplantation), mortality, and quality assessment. The case fatality rate was defined as the proportion of deaths due to any cause among all the patients. Two co-authors independently extracted the data and compared their findings. When necessary, we contacted the article’s author to request further information.

### 2.4. Quality Appraisal

Two investigators independently extracted the data according to the PRISMA guidelines, which comprise a 17-item checklist intended to facilitate the preparation and reporting of a robust systematic review protocol. The checklist is widely used to facilitate the inclusion of relevant protocol information in funding applications and academic manuscripts. In the Supplementary Materials, we summarize a revision of this guideline, which has been updated to address several conceptual and practical advances in the science of systematic reviews.

We adopted the Newcastle–Ottawa Scale for evaluating study quality since its application to evidence-based reviews and meta-analyses produces highly objective results [2]. The Newcastle–Ottawa Scale has several domains: selection, comparability, and outcome measurement. All the studies were independently scored for each domain by the two co-authors. A consensus was reached after classification by each co-author. Studies receiving ≥7 stars were considered high quality, those receiving 4–6 stars were considered moderate quality, and those receiving ≤ 3 were considered poor quality.

### 2.5. Meta-Analysis

The principal summary measures used were pooled prevalence and risk ratio (RR) with a 95% confidence interval (CI). The overall mean effect sizes were estimated using either random-effects or fixed-effects models depending on the included studies’ heterogeneity. Heterogeneity among the estimates was assessed using the *I*^2^ statistic. Accordingly, an *I*^2^ of 0–40% indicates little heterogeneity, 30–60% indicates moderate heterogeneity, 50–90% indicates substantial heterogeneity, and 75–100% indicates considerable heterogeneity. Fixed-effects models were used when the *I*^2^ was <40% [21].

The anticancer treatment group comprised SARS-CoV-2-infected patients with hematologic malignancies receiving anticancer therapy. The non-anticancer treatment group comprised SARS-CoV-2-infected patients with hematologic malignancies not receiving anticancer therapy. The anticancer treatment group was divided into two subgroups: one group comprising patients that received chemotherapy (treatment) and the other comprising patients whose anticancer treatment was delayed (other).

The primary outcome of the meta-analysis was the pooled death risk among patients with hematologic malignancies and COVID-19. The death risks in all the patients and hospitalized patients are reported. Subgroup analyses compared the mortality risk of male or female patients receiving and not receiving anticancer therapy.

We used funnel plots to evaluate publication bias. All the statistical analyses were performed using Review Manager 5.4 software. Statistical significance was defined as a two-tailed *p* < 0.05.

## 3. Results

Figure 1 shows a flowchart of the literature search. We initially screened 13,931 articles using our search criteria. The software removed duplicate references directly. We excluded 13,896 articles after screening their titles and abstracts. We reviewed the full texts of the remaining 35 articles in depth, of which 12 were included in the final analysis (Figure 1). They were published between 2020 and 2022 and included 1200 patients with hematologic malignancies and COVID-19 from 9 countries, and 712 patients were males. The median age of the patients was ~63 years. Zaki et al. (2022) did not report data on age. Regarding the primary diagnoses, seven studies included leukemia and lymphoma, three included only leukemia, and two included only lymphoma. The overall mortality rate was 32.8% [22]. Regarding study quality, most were assigned 6–9 stars, indicating moderate to high quality. The general characteristics of these studies are listed in Table 1.

**Figure 2:**
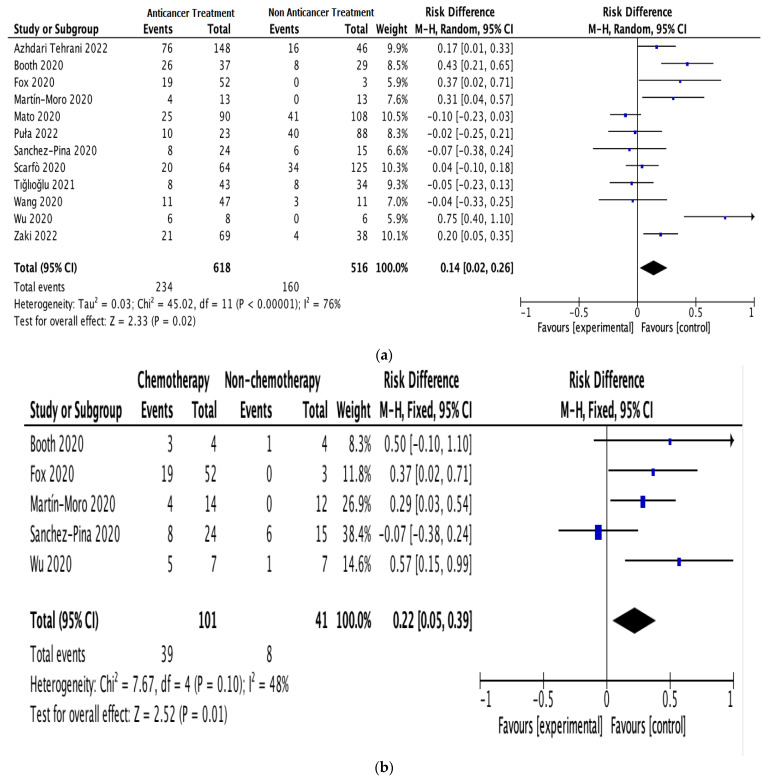

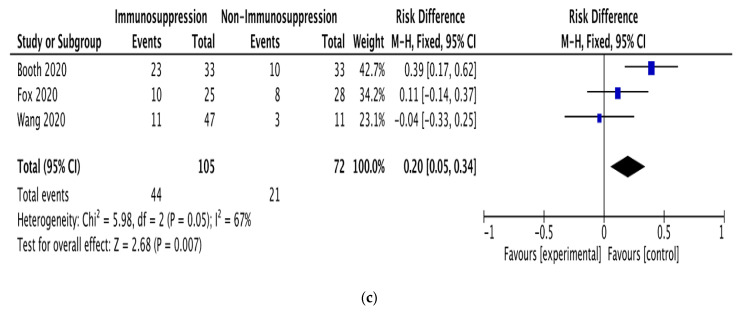
Pooled mortality risk. (**a**) All studies. (**b**) With and without chemotherapy. (**c**) With and without immunosuppressive therapy [19,22,23,24,25,26,27,28,29,30,31,32].

All 12 studies provided information on anticancer therapy, mortality, and cancer exacerbation risk in patients with hematologic malignancies and COVID-19 [19,22,23,24,25,26,27,28,29,30,31,32]. Since there was apparent heterogeneity among these studies (*I*^2^ = 76%, *p* < 0.00001), a random-effects model was used for the assessment [21]. In the anticancer treatment group, 234 out of 618 patients died. In the non-anticancer treatment group, 160 out of 516 patients died. The results indicated a significant correlation between anticancer therapy and mortality risk in patients with hematologic malignancies and COVID-19; the estimated risk difference (RD) between the groups was 0.14 (95% CI: 0.02–0.26; *I*^2^ = 76%; Figure 2a).

Subgroup analyses were performed on the data for patients receiving anticancer therapy, comparing those receiving or not receiving chemotherapy (5 studies; 142 patients) and those receiving or not receiving immunosuppressive therapy (3 studies; 182 patients). The pooled RD for mortality between the patients receiving and not receiving chemotherapy was 0.22 (95% CI: 0.05–0.39; *I*^2^ = 48%; Figure 2b). The pooled RD for mortality between the patients receiving and not receiving immunosuppressive therapy was 0.20 (95% CI: 0.05–0.34; *I*^2^ = 67%; Figure 2c).

Sex-based subgroup analyses were also performed on the data for patients receiving anticancer therapy (2 studies; 33 males and 24 females). The increase in mortality was higher in females (RD = 0.57; 95% CI: 0.29–0.85) than in males (RD = 0.28; 95% CI: 0.04–0.52). There was almost no heterogeneity in the male and female results (*I*^2^ = 0%).

## 4. Discussion

Patients with hematologic malignancies are highly immunocompromised due to their underlying disease and treatments, causing significant concern about their risk of heightened morbidity and mortality from COVID-19 [16]. We report a systematic review and meta-analysis of the literature on the mortality risk of patients with hematologic malignancies and COVID-19 up to 30 September 2022. Our meta-analysis included data on 1200 patients from 9 countries and excluded overlapping studies. The pooled mortality estimate was 32.8%, which is consistent with the 34% mortality estimate (95% CI: 28–39%) reported by a meta-analysis of 34 studies in the PubMed and EMBASE databases published up to 20 August 2020. The meta-analysis included 3240 adult patients from Asia, Europe, and North America [16].

Our study found that mortality risk in adult patients with hematologic malignancies and COVID-19 was higher in those receiving anticancer therapy than in those not receiving anticancer therapy. We found an RD between the patients receiving and not receiving anticancer therapy of 0.14 (95% CI: 0.02–0.26). A recent meta-analysis of studies published up to 20 August 2020 found that the pooled RR for death in patients with hematologic malignancies receiving systemic anticancer therapy (defined as active anticancer therapies, such as cytotoxic chemotherapy, immunotherapy, and targeted agents, and excluding single agent hydroxyurea and steroids) between 28 days and 6 months of a COVID-19 diagnosis was 1.17 (95% CI: 0.83–1.64) [16]. In our study, 234 out of 618 patients receiving anticancer therapy died, whereas 160 out of 516 patients not receiving anticancer therapy died, leading to a crude odds ratio (OR) estimate (unweighted) of 1.22. Although the pooled RR in the previous analysis did not reach statistical significance, our crude OR estimate was close to it and fell within its 95% CI. Therefore, these results can be considered compatible. Another recent meta-analysis on patients with hematologic malignancies and COVID-19 reported severity/mortality ORs associated with surgery, chemotherapy, immunotherapy, and targeted therapy of 1.30 (95% CI: 0.79–2.13), 1.27 (95% CI: 0.95–1.69), 1.20 (95% CI: 0.90–1.61), and 0.92 (95% CI: 0.72–1.19), respectively [33]. Similarly, although these ORs did not reach statistical significance, they are compatible with our study.

Since the COVID-19 pandemic began in 2019, few published articles have examined COVID-19-infected cancer patients with hematologic malignancies. However, in order to ensure that our study was highly accurate, we included only already published articles, removing those that were under review or letters to the editors. In our study, although the pooled overall mortality estimates were mostly from inpatient services, we still observed high heterogeneity (*I*^2^ = 76%).

Oncology patients deserve particular attention during the current pandemic since they are immunocompromised and vulnerable to severe outcomes from SARS-CoV-2 infection due to their underlying malignancy and various anticancer therapies [4]. Our study’s pooled RD for mortality between the patients receiving and not receiving chemotherapy was 0.22 (95% CI: 0.05–0.39), with moderate heterogeneity (*I*^2^ = 48%). Previous studies on SARS-CoV-2-infected cancer patients have shown that those who recently underwent chemotherapy had a higher risk of clinically severe events than those who did not. Our findings on this issue are inconsistent with a meta-analysis that found that cancer patients who received anticancer therapy (including surgery, targeted therapy, radiotherapy, chemotherapy, and immunotherapy) shortly before being diagnosed with COVID-19 did not have an increased risk of exacerbation or mortality [21]. However, this meta-analysis included studies on patients with all cancer types receiving various anti-tumor therapies.

Our pooled RD for mortality between the patients receiving and not receiving immunosuppressive therapy was 0.20 (95% CI: 0.05–0.34), based on studies with substantial heterogeneity (*I*^2^ = 67%). This RD is very close to that for chemotherapy, suggesting that immunosuppressive therapy has a similar effect on the mortality of patients with hematologic malignancy and COVID-19.

COVID-19 is a severe infection in many patients with solid and hematologic malignancies due to their older age, comorbidities, and immunocompromised status [6]. A meta-analysis found that the risk of death in hospitalized patients with hematologic malignancies was 39%, which is comparable to the risk of death for those with solid tumors. However, their risk remained substantially higher than that of the general population. Moreover, their comparable risk of death to patients with solid tumors supports the view that they should not be excluded from more intensive supportive care for COVID-19 based solely on their hematologic diagnosis [16]. Therefore, these patients should be managed appropriately without jeopardizing their curative chances.

Our subgroup analysis showed that among patients with hematologic malignancies and COVID-19 who received anticancer therapy, the increase in mortality among females was more than twice that among males (RD: 0.57 vs. 0.28). It should be noted that there was almost no heterogeneity in the male and female results (*I*^2^ = 0%). To our knowledge, this sex difference has not been previously reported in studies indexed in the three major literature databases we searched. Male–female differences in the immune and endocrine systems might cause different responses to SARS-CoV-2 infection [34]. In our view, the sex effect should be treated with caution due to the observational nature of most of the studies included in our meta-analysis. Nevertheless, studies on sex differences should be conducted in order to identify the underlying mechanisms that might lead to the development of measures to reduce the mortality risk of patients with hematologic malignancies, COVID-19, and both.

Our results suggest that SARS-CoV-2 infection in adult patients with hematologic malignancies is associated with poor outcomes, regardless of their anticancer therapy [11]. Recent studies have also shown that mortality is higher among patients with COVID-19 undergoing anticancer therapy. They recommended that the patient’s cancer burden and overall condition be comprehensively assessed in order to decide whether to proceed with anticancer therapy [9,10], which is consistent with our findings. Notably, anticancer therapy for adult patients with hematologic malignancies and COVID-19 increased the risk of mortality, which is consistent with a previous study [12]. Patients with hematologic malignancies have poorer prognoses than those with solid tumors. They also have high risks of COVID-19 infection, severe post-infection illness, and mortality [15].

Although molecular-targeted therapy rarely impairs a patient’s immunity, those receiving maintenance molecular-targeted therapy all have advanced disease, potentially explaining their increased risk of death. Patients with hematologic malignancies had poorer prognoses than those with solid tumors [34]. In addition to the inherent differences between hematologic and solid malignancies, the prevalence of cancer in patients with COVID-19 is uncertain. Previous studies from China have reported that 1–2% of COVID-19 patients had cancer. In contrast, a study from the United States reported that 6% of hospitalized patients with COVID-19 had cancer [21,32]. It is important to consider that patients with hematological malignancies are immunocompromised. Our study showed an increased risk of serious COVID-19-related events in patients with hematological malignancies than in patients without cancer.

It has been reported that 39% of COVID-19 patients with cancer experienced severe events, including death, during their COVID-19 course, compared to only 8% of COVID-19 patients without cancer. The more aggressive clinical course of cancer patients with COVID-19 may reflect immunosuppression due to chemotherapy, radiotherapy, or immunosuppressive therapy; increased comorbidities; or lung invasion by the primary tumor or metastases [35]. In addition, SARS-CoV-2 might affect hematopoiesis and cause cytopenia, including lymphocytopenia [36]. One study proposed that viral spike protein cytotoxicity damages hematopoietic stem cells [37]. While the recent Omicron variant appears to have unique features and stronger interferon responses to live attenuated viruses, which may enhance chimerism-mediated immunotherapy, it does not appear to increase mortality [33]. We suggest that future research adhere to key methodological principles, such as controlling for the factors most likely to influence the study results. Further studies should investigate topics to aid clinical practice and decision-making. The definition of outcomes should be standardized to aid in comparing patients with hematologic malignancies in different studies.

Our meta-analysis results provide precise and reliable evidence for developing practical guidelines and managing COVID-19 in patients with hematologic malignancies. However, it also had some limitations. First, we only included data reported in the literature and did not contact authors for unreported data. Second, it included studies that were mainly retrospective and potentially subject to inherent bias. Third, due to differences in the age and treatment plans of cancer patients, it is difficult to achieve a balance in baseline data among study subjects, potentially affecting the accuracy of out results. Fourth, it included data from the SARS-CoV-2 pandemic’s pre-vaccination and antiviral drug stages. Therefore, our observations may have been influenced by vaccines and active treatments.

Lower respiratory tract diseases caused by SARS-CoV-2 infection in patients with hematologic malignancies are associated with high rates of oxygen use and mortality. Hospitalized patients with cancer and COVID-19 had a high case fatality rate [34]. COVID-19 has caused unprecedented societal turmoil and is still ongoing, transforming healthcare provision globally. Acknowledging the challenges this pandemic poses in caring for particularly vulnerable patients, such as those with hematologic malignancies, is imperative. Evolving treatment strategies and novel approaches that address the needs of oncology patients in the context of the pandemic have been discussed elsewhere [4].

## 5. Conclusions

Patients receiving chemotherapy might develop long-lasting myelosuppression and impaired immunity [18]. Our meta-analysis showed that, among patients with hematologic malignancies and COVID-19, those receiving anticancer therapy had a higher mortality risk, regardless of sex. In particular, females had a higher mortality risk than males. These results suggest that caution should be exercised when administering anticancer therapy to patients with hematologic malignancies and COVID-19. Patients must be informed about COVID-19 symptoms, trained in proper hand-washing hygiene practices, and given guidance on using personal protective equipment whenever they go out in public or visit the hospital.

## Figures and Tables

**Table 1:** Summary of the 12 studies included in the meta-analysis.

Studies/Years	Region	Number of Patients	Age (Year)	Sex (Male)	Anticancer Therapy	Mortality (Event/Total)	Newcastle–Ottawa
Azhdari Tehrani, 2022 [23]	Iran	194	44 (15–81)	135	Chemotherapy vs. no treatment	47.4% (92/194)	7
Booth, 2020 [24]	UK	66	73 (63–81)	41	Immunosuppressive or chemotherapy in the last 3 months vs. no treatment	51.5% (34/66)	7
Fox, 2020 [25]	UK	55	63 (23–88)	37	Immunosuppressive or chemotherapy vs. no treatment	34.6% (19/55)	7
Martín-Moro, 2020 [26]	China	34	72.5 (35–94)	19	Chemotherapy vs. no treatment	32.4% (11/34)	9
Mato, 2020 [19]	USA	178	71 (41–98)	110	Immunosuppressive or chemotherapy vs. no treatment	37.1% (66/178)	9
Puła, 2022 [27]	Poland	175	68 (37–87)	70	Targeted therapy vs. no treatment	26.6% (50/188)	9
Sanchez-Pina, 2020 [28]	Spain	39	64.7 (36–88)	23	Chemotherapy vs. no treatment	35.8% (14/39)	9
Scarfò, 2020 [29]	Italy	190	72 (48–94)	126	Chemotherapy vs. no treatment	29.5 (56/190)	6
Tığlıoğlu, 2021 [30]	Turkey	77	60 (27–93)	40	Immunosuppressive or chemotherapy vs. no treatment	20.7% (16/77)	7
Wang, 2020 [31]	USA	58	67	30	Immunosuppressive vs. no treatment	24.0% (14/58)	7
Wu, 2020 [32]	China	14	36.5 (14–68)	9	Immunosuppressive or chemotherapy vs. no treatment	42.8% (6/14)	7
Zaki, 2022 [22]	Pakistan	107	NA *	72	Immunosuppressive or chemotherapy vs. no treatment	23.4% (25/107)	9

* NA: not available.

## Data Availability

The data presented in this study are available on request from the corresponding author.

## Supplementary Materials

The following supporting information can be downloaded at: https://www.mdpi.com/article/10.3390/life13020381/s1. Reference [38] has been cited in Supplementary Materials.
